# 625 nm Light Irradiation Prevented MC3T3-E1 Cells from Accumulation of Misfolded Proteins via ROS and ATP Production

**DOI:** 10.3390/ijms24119257

**Published:** 2023-05-25

**Authors:** Wenqi Fu, Yeong-Gwan Im, Byunggook Kim, Ok-Su Kim, Ying Yang, Jianan Song, Danyang Liu, Siyu Zhu, Jae-Seok Kang, Okjoon Kim

**Affiliations:** 1 Department of Oral Pathology, School of Dentistry, Chonnam National University, Gwangju 61186, Republic of Korea; 2 Department of Oral Medicine, School of Dentistry, Chonnam National University, Gwangju 61186, Republic of Korea; 3 Department of Periodontology, School of Dentistry, Chonnam National University, Gwangju 61186, Republic of Korea

**Keywords:** light-emitting diode irradiation (LEDI), binding immunoglobulin protein (BiP), reactive oxygen species (ROS), adenosine triphosphate (ATP), endoplasmic reticulum (ER) stress, bone

## Abstract

Osteoblasts must acquire a considerable capacity for folding unfolded and misfolded proteins (MPs) to produce large amounts of extracellular matrix proteins and maintain bone homeostasis. MP accumulation contributes to cellular apoptosis and bone disorders. Photobiomodulation therapy has been used to treat bone diseases, but the effects of decreasing MPs with photobiomodulation remain unclear. In this study, we explored the efficacy of 625 nm light-emitting diode irradiation (LEDI) to reduce MPs in tunicamycin (TM) induced-MC3T3-E1 cells. Binding immunoglobulin protein (BiP), an adenosine triphosphate (ATP)-dependent chaperone, is used to evaluate the capacity of folding MPs. The results revealed that pretreatment with 625 nm LEDI (Pre-IR) induced reactive oxygen species (ROS) production, leading to the increased chaperone BiP through the inositol-requiring enzyme 1 (IRE1)/X-box binding protein 1s (XBP-1s) pathway, and then restoration of collagen type I (COL-I) and osteopontin (OPN) expression relieving cell apoptosis. Furthermore, the translocation of BiP into the endoplasmic reticulum (ER) lumen might be followed by a high level of ATP production. Taken together, these results suggest that Pre-IR could be beneficial to prevent MP accumulation through ROS and ATP in TM-induced MC3T3-E1cells.

## 1. Introduction

Numerous genetic and environmental factors disrupt protein folding efficacy, leading to the accumulation of misfolded proteins (MPs) within the endoplasmic reticulum (ER), a state called ER stress [1]. ER stress activates the sensors’ protein kinase RNA-like ER kinase (PERK), inositol-requiring enzyme 1 (IRE1), and activating transcription factor 6 (ATF6), representing the three branches of the unfolded protein response (UPR). All three UPR sensors first attempt to realign protein-folding demand and capacity back to homeostasis. However, prolonged ER stress is harmful to the cell and promotes cell death. ER stress is involved in various pathological processes of bone tissue, such as periodontitis, osteoporosis, and arthritis [2,3,4]. Thus, it is beneficial for treating bone disease by reducing the accumulation of MPs in the ER.

Photobiomodulation involves the use of red or near-infrared light at low power densities to produce a beneficial effect on cells or tissues [5]. It has been used to accelerate damaged bone tissue restoration, such as healing of the extraction socket and distraction osteogenesis [6,7]. This treatment might be associated with the functions of cytoprotection [8]. However, the detailed mechanisms remain unclear.

Red light at wavelengths 600 nm to 760 nm is capable of influencing several activities in living cells and tissues. The chemical effects of these wavelengths stem from the absorption of photons by chromophores in the mitochondria and its reactions with respiratory oxygen [9]. As a result, reactive oxygen species (ROS) are produced and are followed by a cascade of biological effects, such as protein expression and metabolic rearrangements [10]. The concept of hormesis has been applied to ROS [11]. Low ROS levels have a large impact on stress resistance [12]. The experimental evidence suggests that pretreatment with photobiomodulation can increase ROS in cells while protecting the stressed cells from cell death [13]. However, the role of ROS in ER stress is not yet fully understood.

Binding immunoglobulin protein (BiP), as a member of the heat shock protein 70 (hsp70) family in the ER, is the master protein responsible for refolding MPs to maintain homeostasis [14]. In addition to its role as a major ER chaperone, BiP has been suggested to be a switch to regulate UPR activation [14]. In unstressed cells, BiP binds with the stress sensor to form a BiP sensor complex system and inactivates the UPR [15]. Upon ER stress, BiP is released from the complex by its high affinity for MPs, and the activated sensors initiate signaling cascades, leading to the transcriptional induction of chaperone genes such as BiP [16]. The abundance of BiP, which normally represents about 0.5% of the human proteome, increases up to 100-fold upon ER stress [17,18]. Thus, BiP is a marker of MP accumulation. However, BiP is also induced to prevent cells from ER stress [19,20]. Our previous study revealed that photobiomodulation served a protective role in ER stress induced-cells [21]. Together, the interaction between photobiomodulation-induced BiP and ER stress sensors remains unclear.

BiP is divided into an adenosine triphosphate (ATP) binding domain (ABD) and a substrate-binding domain (SBD) [22]. BiP has greater sensitivity for MPs in the ATP-bound state via conformational change [23]. Photobiomodulation can increase cellular ATP generation from oxygen and pyruvate [24]. Thus, the function of photobiomodulation-induced ATP in regulating BiP location remains to be explored.

The stress-relieving effect of photobiomodulation suggests that it protects cells from cellular disorders. Tunicamycin (TM) induces ER stress by inhibiting protein glycosylation, resulting in MP accumulation in the ER [25]. Decreasing ER stress has a positive effect on treating bone disease [3]. To explore the mechanism of the photobiomodulation in ER stress, we exposed MC3T3-E1 cells to 625 nm light-emitting diode irradiation (LEDI) before treating cells with the TM and assessed the effects of LEDI on MP accumulation. We discovered that LEDI was beneficial for relieving ER stress and restoring bone formation in MC3T3-E1 cells.

## 2. Results

### 2.1. Pre-IR Treatment Recovered Cell Viability and Increased BiP Expression

**Figure 1:**
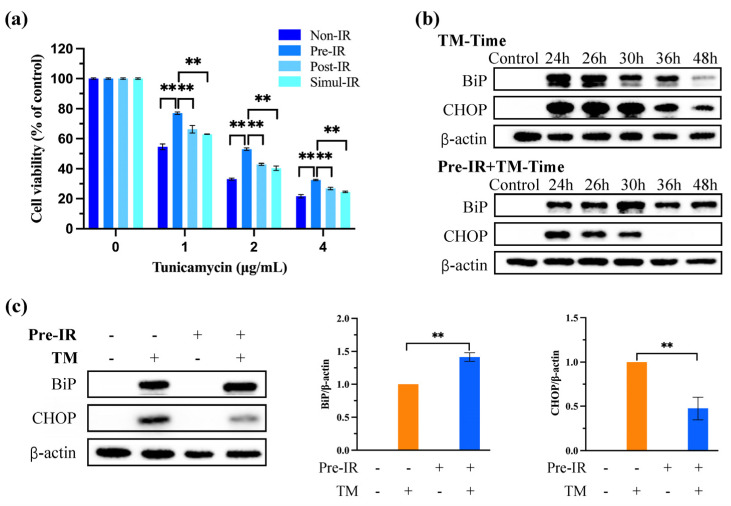
Pre-IR treatment-recovered cell viability and increased BiP expression. (**a**) Cell viability of MC3T3-E1 cells measured with EZ-Cytox staining after various doses (0, 1, 2, and 4 μg/mL) of TM treatment for 24 h with or without Pre-IR, Post-IR, and Simul-IR. (**b**) The expression of BiP and CHOP detected at 0 h (24 h), 2 h (26 h), 6 h (30 h), 12 h (36 h), and 24 h (48 h) after 1 μg/mL TM for 24 h using western blotting assay. (**c**) Changes in BiP and CHOP expression at 48 h detected using western blotting assay. Quantification of the intensities of bands relative to those of β-actin is shown. ** *p* < 0.01, compared with the control group.

TM was used to establish an ER stress model in MC3T3-E1 cells, which induced cell death. The cells were treated with TM at 0, 1, 2, and 4 μg/mL before (Pre-IR), after (Post-IR), or during (Simul-IR) irradiation with 625 nm LEDI for one hour. The controls (Non-IR) were only treated with TM without irradiation. As shown in Figure 1a, the cell viability decreased depending on the TM dose, and about 50% of cells died with treatment with 1μg/mL TM, which was chosen for further study. Compared with Post-IR and Simul-IR, Pre-IR significantly restored cell viability even with TM. Therefore, the mechanism of the preventative effect of Pre-IR on ER stress was furtherly explored.

To determine whether Pre-IR reduced ER stress, the ER stress-associated proteins, BiP and C/EBP homologous protein (CHOP), were measured time-dependently in the TM and Pre-IR+TM groups. The results revealed that BiP and CHOP were expressed differently after Pre-IR (Figure 1b). In the TM group, BiP and CHOP gradually decreased with time. Interestingly, Pre-IR maintained BiP at a high level within 48 h, but CHOP expression decreased sharply after 30 h, which may be associated with the accelerated reduction of MPs. To furtherly confirm these results, we detected the expressions of BiP and CHOP at 48 h. As shown in Figure 1c, compared with the TM group, Pre-IR increased BiP and decreased CHOP simultaneously. These results suggested that Pre-IR recovered the cell viability and promoted BiP expression.

### 2.2. 625 nm LEDI Promoted the Expression of BiP via ROS Production

**Figure 2:**
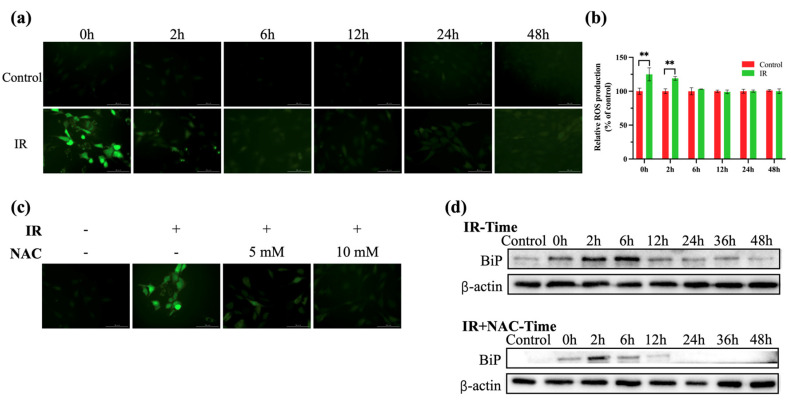
625 nm LEDI promoted the expression of BiP via ROS production. (**a**) ROS production assessed using DCF-DA staining at 0 h, 2 h, 6 h, 12 h, 24 h, and 48 h after LEDI treatment for one hour. (**b**) Quantification of ROS production assessed using DCF-DA staining. (**c**) ROS production assessed with DCF-DA staining immediately after LEDI treatment for one hour with or without NAC pretreatment. (**d**) BiP expression detected with western blotting assay at 0 h, 2 h, 6 h, 12 h, 24 h, and 48 h after LEDI for one hour with or without NAC pretreatment. ** *p* < 0.01.

To determine the mechanism behind the preventative effect of 625 nm LEDI in MC3T3-E1 cells, the levels of ROS production and BiP expression were measured. After exposure of cells to 625 nm LEDI for one hour, the 2′,7′-dichlorodihydrofluorescein diacetate (DCF-DA) fluorescence intensity was increased at the end of the irradiation, indicating increased ROS production (Figure 2a,b). As shown in Figure 2c, compared with treating 5 mM N-acetyl-L-cysteine (NAC), 10 mM NAC significantly scavenged LEDI-induced ROS production, which was chosen for further study.

The effect of ROS on BiP expression was evaluated with western blotting. As shown in Figure 2d, BiP gradually increased for six hours, then returned to its original level. Moreover, BiP expression was decreased at all time points during NAC treatment. Together, these data demonstrated that ROS induced with 625 nm LEDI upregulated BiP expression.

### 2.3. The Effects of Pre-IR on ER Stress Sensors

**Figure 3:**
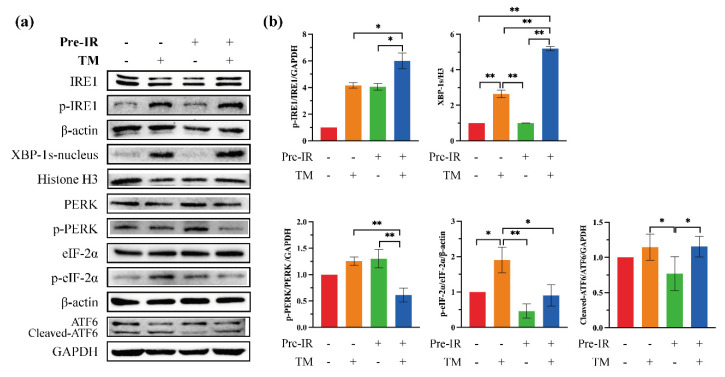
The effects of Pre-IR on ER stress sensors. (**a**) The expression of ER stress-related proteins detected with western blotting assay. (**b**) Quantification of the intensities of bands relative to those of β-actin/GAPDH/H3 is shown. * *p* < 0.05; ** *p* < 0.01, compared with the control group.

In order to explore the effects of Pre-IR on ER stress, three stress sensors in the ER membrane and associated transcription factors were investigated. The results of western blotting are shown in Figure 3a, and their quantification is shown in Figure 3b. Pre-IR promoted IRE1 phosphorylation and translocation of X-box binding protein 1s (XBP-1s) into the nucleus, while the expressions of p-PERK and phosphorylation of the eukaryotic initiation factor 2α (p-eIF-2α) were decreased. There was no obvious change in cleaved-ATF6. These results indicate that Pre-IR upregulated the IRE1/XBP-1s pathway and downregulated the PERK/eIF-2α pathway.

### 2.4. Pre-IR Upregulated the Expression of BiP via the ROS Production

**Figure 4:**
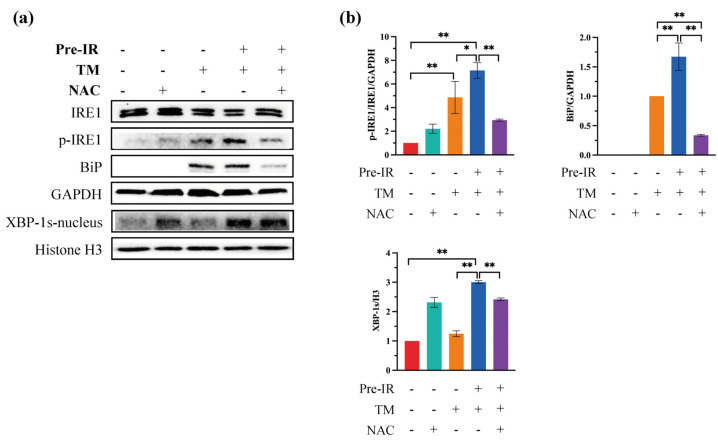
Pre-IR upregulated the expression of BiP via the ROS production. (**a**) The expression of IRE1, p-IRE1, BiP, and XBP-1s was detected with western blotting assay. (**b**) Quantification of the intensities of bands relative to those of GAPDH/H3 is shown.* *p* < 0.05; ** *p* < 0.01, compared with the control group.

To further determine whether Pre-IR regulates BiP through ROS and explore the potential mechanism, NAC was used to scavenge ROS, and the expression of p-IRE1/IRE1, XBP-1s, and BiP was detected with western blotting. Results are shown in Figure 4a, and these data are quantified in Figure 4b. Pre-IR significantly increased the expression of p-IRE1 and BiP and promoted the translocation of XBP-1s into the nucleus. However, this trend was blocked with treatment with NAC. These results demonstrate that Pre-IR-induced ROS upregulated BiP expression through the IRE1/XBP-1s pathway.

### 2.5. TM-Induced Apoptosis and the Decrease in Osteogenic Gene Expression Were Prevented by ROS Production

**Figure 5:**
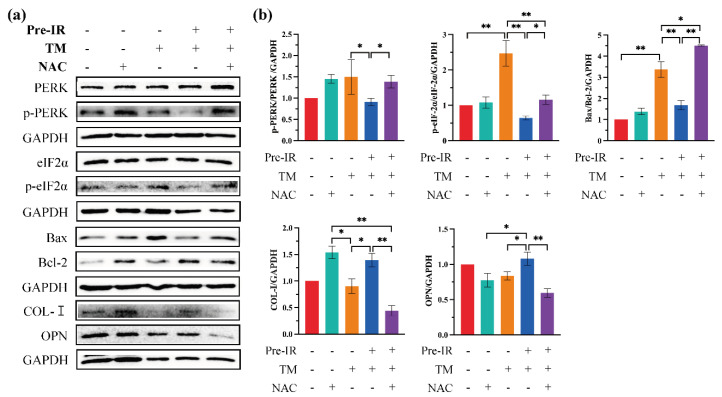
TM-induced apoptosis and the decrease in osteogenic protein expression were prevented via ROS production. (**a**) The expression of eIF2α, p-eIF2α, Bax, Bcl-2, COL-I, and OPN detected with western blotting assay. (**b**) Quantification of the intensities of bands relative to those of GAPDH is shown. * *p* < 0.05; ** *p* < 0.01, compared with the control group.

Prolonged ER stress disrupts protein homeostasis, which results in cell apoptosis. To confirm whether the efficacy of protein folding was restored with Pre-IR, apoptosis proteins (B-cell lymphoma protein 2 (Bcl-2)-associated X (Bax), Bcl-2) and bone-formation-related proteins (collagen type I (COL-I), osteopontin (OPN)) were evaluated with western blotting. Results are shown in Figure 5a, and these data are quantified in Figure 5b. Phosphorylation of PERK and eIF2α was observed in the TM group, which might indicate that the global protein translation is reduced. Consistent with these results, the expression of anti-apoptotic Bcl-2 and osteogenic COL-I and OPN was decreased with TM and prevented with Pre-IR. COL-I and OPN were significantly increased, and the ratio of Bax/Bcl-2 was decreased in the Pre-IR group, compared with the TM group. However, the preventative effect of Pre-IR was suppressed with NAC. Taken together, Pre-IR prevented apoptotic cell death and the decrease in osteogenic gene expression via ROS production.

### 2.6. Pre-IR Increased ATP Generation and BiP Translocation into the ER Lumen

**Figure 6:**
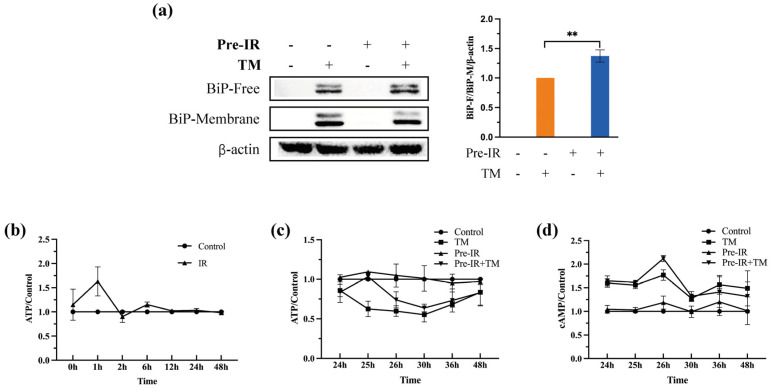
Pre-IR increased the ATP generation and BiP translocation into the ER lumen with a high ATP level. (**a**) The expression of BiP located in the ER lumen and membrane detected through western blotting assay. Quantification of the intensities of bands relative to those of β-action is shown. (**b**,**c**) Time course of intracellular ATP level detected through luminescence assay. (**d**) Time course of intracellular cAMP level detected through chemiluminescent assay. Quantification with luminescence/chemiluminescent plate reader of the relative light unit values per mg cell protein from the BCA assay. ** *p* < 0.01, compared with the control group.

BiP plays different roles depending on its subcellular location. To further determine the mechanism of Pre-IR in preventing ER stress through BiP, cytosol and membrane protein were separated and evaluated with western blotting. As shown in Figure 6a, the BiP-Free/BiP-Membrane ratio was increased in Pre-IR compared with TM. These results indicate that Pre-IR promoted the translocation of BiP into the ER lumen under ER stress, where BiP bound with MP substrates and resulted in MP refolding.

BiP is a kind of ATP-dependent chaperone, and ATP primes BiP to engage MPs, thereby facilitating the dissociation from the cell membrane [20]. The content of ATP and cyclic adenosine 3′,5′-monophosphate (cAMP) was detected and normalized by the protein content, which was detected via bicinchoninic acid (BCA) assay. An amount of 625 nm LEDI promoted ATP production for one hour (Figure 6b). Further, as shown in Figure 6c, the ATP content of the Pre-IR group was higher than that of the TM group at all time points within 48 h, which was confirmed with the results of cAMP (Figure 6d). These findings indicate that Pre-IR accelerated MP refolding through the translocation of BiP into the ER lumen under a high ATP level.

## 3. Discussion

Bone is primarily composed of extracellular matrix (ECM) proteins and calcium phosphate [26]. During bone remodeling, osteoblasts produce a large number of secreted proteins to synthesize the ECM, and these proteins must be quality controlled [2,27]. ER stress, an important part of quality control systems, participates in the physiological process of osteogenesis, while unmanageable ER stress results in the disruption of bone homeostasis [28,29]. Previous studies from our laboratory have shown that Pre-IR exerted a positive effect on bone formation by protecting MC3T3-E1 cells from ER stress [21]. However, the potential mechanisms have not yet been understood. In the present study, we further explored the preventative effect of Pre-IR on ER stress in MC3T3-E1 cells.

We established an ER stress model by treating MC3T3-E1 cells with TM. As shown in Figure 1a, compared with Post-IR and Simul-IR, Pre-IR significantly restored the decrease in cell viability caused by TM, which is consistent with our previous results [21]. Commonly, BiP and CHOP are regarded as ER stress markers, which are used to evaluate the severity of stress. However, various studies revealed that increased BiP expression protected cells from ER stress [19,20]. In this study, we also found that BiP was sustained at a high level via Pre-IR treatment, while CHOP was reduced (Figure 1b). Consistent with these results, Figure 1c showed that BiP expression was increased; however, CHOP expression was decreased with Pre-IR at 48 h compared with the TM group. These results may be related to the synthesis and metabolism of BiP. The half-life of BiP ranges from 2 to over 24 h, including in cells experiencing ER stress [18,30]. BiP degraded over time in the TM group, while Pre-IR promoted the increase in BiP expression and thereby restored cell viability.

Experimental evidence supports the theory that photobiomodulation triggers the production of ROS and ATP and then initiates signaling cascades that play roles in cytoprotection [8,24]. The results shown in Figure 2a,b, and Figure 6b are consistent with this theory. It is well known that ROS has complex influences on cells depending on the concentration. A mild increase in ROS triggers various cellular molecules that allow cells to resist stress [13]. As shown in Figure 2d, ROS, as a kind of stimulus, induced the expression of BiP, which plays a beneficial effect in increasing protein-folding capacity.

We further explored the target of Pre-IR-induced ROS on ER stress. Three stress sensors on the ER membrane (PERK, ATF6, and IRE1) operate in parallel and use unique mechanisms of signal transduction [1]. When active, PERK phosphorylates eIF2α, which inhibits eIF2α activity and causes global protein translation attenuation [31]. IRE1 phosphorylation results in the stimulation of endoribonuclease activity, which splices mRNA of XBP1 to form the homeostatic transcription factor XBP1s. XBP1s translocates into the nucleus and increases the transcription of chaperone genes, such as BiP, which increases protein folding capacity [32]. Thus, the IRE1/XBP-1s pathway plays a crucial role in alleviating the burden of MP accumulation within the ER [33]. Figure 3 shows the effects of Pre-IR on ER stress sensors. Consistent with Figure 1c results, Pre-IR induced the activation of p-IRE1 and promoted the translocation of XBP-1s into the nucleus upstream of BiP. To confirm the role of ROS in this process, NAC was used to scavenge ROS, and related proteins were detected with western blotting assay. As shown in Figure 4, the increase of p-IRE1 and XBP-1s, induced with Pre-IR, was reduced with NAC. Simultaneously, the expression of BiP was decreased. According to these results, we proved that Pre-IR-induced ROS upregulated the expression of BiP through the IRE1/XBP-1s pathway but not the ATF6 pathway. Moreover, phosphorylated eIF2α results in protein translation attenuation, giving the cell extra time to attempt to fold the backlog of proteins already present in the ER lumen. Although a temporary pause in protein translation can be beneficial for cells under ER stress, a protracted block in translation from a sustained PERK/eIF2α signaling pathway has harmful effects on essential protein synthesis for cell homeostasis [34]. Osteoblasts secrete COL-I as the major constituent of the ECM, and various osteogenic markers, such as OPN, regulate matrix mineralization [35]. Based on the results of Figure 5, we found that Pre-IR restored the expression of COL-I and OPN under ER stress, compared with the TM group, but this positive effect was decreased through scavenging ROS. Similarly, the expression of the anti-apoptosis protein, Bcl-2, also followed this pattern, which is consistent with the previous evidence [36]. Together, the results demonstrate that Pre-IR was beneficial for promoting the efficacy of MP refolding and preventing the harmful effect of ER stress.

**Figure 7:**
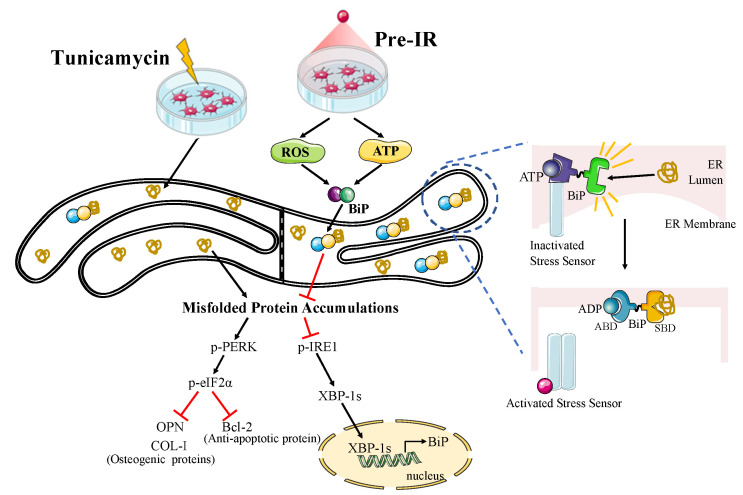
Scheme illustration of the protective effect of 625 nm LEDI on TM-induced MC3T3-E1 cells. Pretreatment with 625 nm LEDI (Pre-IR) protected cells from misfolded protein (MP) accumulation via ROS production and ATP generation. TM was used to establish an ER stress model. Prolonged ER stress sustainedly activates the PERK/eIF2α pathway, resulting in the translation of essential proteins being blocked and then inducing cell apoptosis. However, Pre-IR-induced ROS, as a mild stimulus, increased chaperone BiP expression through IRE1/XBP-1s pathway, which made cells stronger to resist ER stress. On the other hand, Pre-IR-induced ATP facilitated BiP dissociation from the ER membrane into the ER lumen, where it assisted in refolding MPs. Together, Pre-IR could be beneficial to prevent MP accumulation via ROS and ATP in TM-induced MC3T3-E1 cells. (→ means promotion; 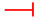 means inhibition.).

The role of BiP is dependent on its subcellular location. In normal conditions, BiP binds with ER stress sensors to form a BiP-sensor complex in the ER membrane; in stress conditions, BiP dissociates from sensors and is released into the ER lumen, where it connects with MPs and assists them in refolding [37]. In this study, we separated the cell membrane and cytosolic proteins and measured the expression of BiP. As shown in Figure 6a, the ratio of BiP-Free/BiP-Membrane was significantly increased with Pre-IR treatment. To explain this phenomenon, we investigated the levels of ATP and cAMP at different time points. A previous study revealed that a high level of ATP facilitated BiP dissociation from the ER membrane into the ER lumen, where it assists in refolding MPs [23]. Figure 6c shows that the levels of ATP were higher in the Pre-IR group than in the TM group. These results also confirmed that the capacity of ATP generation was restored, meaning the cellular stress condition was partially relieved with Pre-IR. cAMP, a molecule derived from ATP, is important in many biological processes [38]. Various studies revealed that cAMP generation prevented cells from ER stress and apoptosis [39,40]. Again, in the present study, compared with the TM group, the cAMP level was higher in the Pre-IR group at all time points, which further confirmed the ATP results and was consistent with previous studies (Figure 6d). These data suggest that ATP might promote BiP translocation into the ER lumen induced with Per-IR in MC3T3-E1 cells. Taken together, as shown in Figure 7, our results suggest that the pretreatment of 625 nm LEDI regulated the expression and condition of BiP through the ROS-involved signaling pathway and ATP generation and prevented TM-induced ER stress from accumulating MPs in MC3T3-E1 cells.

## 4. Materials and Methods

### 4.1. Cell Culture

MC3T3-E1 cells were cultured in α-modified Eagle’s medium (α-MEM; Gibco, BRL, Gaithersburg, MD, USA) supplemented with 10% fetal bovine serum (FBS; Atlas Biologicals, Seoul, Republic of Korea) and 1% penicillin and streptomycin (Wel gene, Gyeongsangbuk-do, Republic of Korea) at 37 °C in a humid 5% CO_2_ atmosphere. The medium was changed every 2–3 days, and the cells were passaged when they reached 70–80% confluence.

TM was used to induce ER stress and was dissolved in dimethyl sulfoxide (DMSO) to make a 1 mg/mL stock solution. All stock solutions were stored at −20 °C.

To scavenge ROS, the cells were pretreated with 5 or 10 mM N-acetyl-L-cysteine (NAC; Sigma, MO, USA) for 6 h.

### 4.2. Irradiation Treatment

The manufactured LED (U-JIN LED, Gwangju, Republic of Korea) irradiation system was installed in an incubator with a humid 5% CO_2_ atmosphere at 37 °C. Based on our previous study [21,41], the LEDI was set to 625 nm wavelength, 5 mW/cm^2^, and 18 J/cm^2^. The cells were irradiated for one hour, and the distance between cells and light was 25 mm.

### 4.3. Cell Viability Assay

The cell viability was evaluated using the EZ-Cytox assay kit (DoGenBio, Seoul, Republic of Korea). Briefly, 5 × 10^3^/well MC3T3-E1 cells were seeded in a 96-well plate in at least triplicate wells for each group. The cells were cultured for 24 h to allow for attachment before treating cells. According to the manufacturer, after the original culture medium was removed, the cells were incubated with the new medium (100 μL) containing 10 μL of Ez-Cytox reagent at 37 °C for one hour. The absorbance was measured at 450 nm using a microplate reader (BioTek, El Segundo, CA, USA).

### 4.4. ROS Detection

The intracellular ROS level was determined via a fluorescent probe, DCF-DA (Sigma, St. Louis, MO, USA) assay. The cells were seeded at 5 × 10^3^/well in a 96-well plate. The cells were irradiated with 625 nm LEDI or not after culturing for 24 h and then treated with 25 μM DCF-DA 0, 2, 6, 12, 24, and 48 h after irradiation. After incubating at 37 °C for 20 min, the cells were washed twice with 1× phosphate-buffered saline (PBS), followed by fluorescence using fluorescence microscopy (Lionheart^TM^ FX, Winooski, VT, USA) and quantification of fluorescence intensity using a luminescence microplate reader (BioTek, CA, USA) with 488 nm excitation and 535 nm emission filters.

### 4.5. Intracellular ATP Assay

The ATP Determination Kit (Invitrogen, Oxford, UK) was used for the quantified measurement of intracellular ATP level. The cells were lysed with lysis buffer and collected. Lysates (10 μL) were transferred to 96-well plates for measuring protein concentration by using the BCA protein assay kit (Thermo Fisher Scientific, Hanover Park, IL, USA). Lysates (10 μL) were transferred to 96-well plates with white walls, and the standard reaction solution (90 μL) was added for ATP measurement. The luminescence signal was measured with a luminescence microplate reader.

### 4.6. Intracellular Cyclic AMP Assay

The Cyclic AMP Assay Kit (Cell Signaling Technology, Danvers, MA, USA) was used for the quantified measurement of intracellular cyclic cAMP level. The cells were lysed with lysis buffer and collected. The assay was completed following the manufacturer’s instructions. The absorbance was measured at 450 nm with a luminescence microplate reader. The cAMP standard curve was used to calculate the absolute amount of cAMP in the test samples.

### 4.7. Western Blotting Analysis

Western blotting was performed as described previously with minor modifications [42]. Briefly, the total proteins were extracted from cells using radioimmunoprecipitation assay (RIPA) lysis buffer containing phenylmethylsulfonyl fluoride (PMSF), PIC, and p-PIC (TaKaRa, Tokyo, Japan), membrane, and cytosolic proteins were extracted via Mem-PER Plus Membrane Protein Extraction Kit (Thermo Fisher Scientific, IL, USA) and quantified with BCA assay. Then, the proteins were separated with SDS-PAGE gel electrophoresis and transferred to polyvinylidene difluoride (PVDF) membranes (cytiva, Seoul, Republic of Korea), followed by blocking in 5% milk-Tris-buffered saline (TBST) buffer for one hour at room temperature. The membranes were incubated overnight at 4 °C with the following primary antibodies: BiP (1:1000), CHOP (1:1000), XBP-1s (1:1000), eIF-2α (1:1000), p-eIF-2α (1:1000), β-actin (1:1000), Histone H3 (1:1000) from Cell Signaling Technology; IRE1 (1:1000), p-IRE1 (1:1000), COL-I (1:1000), OPN (1:1000) from Abcam (Abcam, Cambridge, UK); PERK (1:500), p-PERK (1:500), ATF-6α (1:500), Bax (1:500), Bcl-2 (1:500), GAPDH (1:500) from Santa Cruz Biotechnology (Santa Cruz Biotechnology, Santa Cruz, CA, USA). After primary antibody incubation, the appropriate horseradish peroxidase (HRP) conjugated secondary antibody (1:10,000, Thermo Fisher Scientific, IL, USA) was used. Protein bands were visualized using the Bio-Rad XRS chemiluminescence detection system (Bio-Rad, Hercules, MA, USA).

### 4.8. Statistical Analysis

Results were expressed as mean ± standard deviation (SD). The statistical analyses were performed with one-way ANOVA using SPSS. The following p-values were considered significant: * *p* < 0.05, ** *p* < 0.01. All experiments were repeated in triplicate.

## Data Availability

All data are contained in the article.

## Abbreviations

ABD: adenosine triphosphate (ATP)-binding domain; ATF6: activating transcription factor 6; BiP: binding immunoglobulin protein; BCA: bicinchoninic acid; Bax: B-cell lymphoma protein 2 (Bcl-2)-associated X; cAMP: cyclic adenosine 3′,5′-monophosphate; CHOP: C/EBP homologous protein; COL-I: collagen type I; DCF-DA: 2′,7′-dichlorodihydrofluorescein diacetate; ECM: extracellular matrix; eIF-2α: eukaryotic initiation factor 2α; ER: endoplasmic reticulum; hsp70: heat shock protein 70; IRE1: inositol-requiring enzyme 1; LEDI: light-emitting diode irradiation; MPs: misfolded proteins; NAC: N-acetyl-L-cysteine; OPN: osteopontin; PERK: protein kinase RNA-like ER kinase; ROS: reactive oxygen species; SBD: substrate-binding domain; TM: tunicamycin; UPR: unfolded protein response; XBP-1s: X-box binding protein 1s.
